# 

**Published:** 1875-03

**Authors:** 


The Relations of the Nervous System to Diseases of the Skin. By L. Duncan Bulkley, A.M., M.D. pp. 30.

---

Note on the Action of Lobelina on the Circulation. By I. Ott, M.D., of Easton, Pa.; pp. 7.

---

Report of 105 Cases of Operation for Cataract. By B. Joy Jeffries, A.M., M.D., Harv. Boston, Mass.; pp. 15.

---

Report of the Committee on Sick, Hospital and Sanitary Measures, of the Chicago Relief and Aid Society; pp. 63.

---

Report of the Health Officer of the City and County of San Francisco; 1874; pp. 71.

---

Report of the Vaccine Department of the New York Dispensary, for 1874. By Frank P. Foster, M.D.; pp. 15.

---

### Editorial.

### INDEX TO VOLUME XII.

Abdomen and chest, penetrating wound of .................... 307
Abortion, treatment of..79, 152, 688
Abscess, chronic pelvic. ....... 212
Pelvic, opening in colon.... 462
Treatment of............... 99
Acid salicylic.................. 758
Address Valedic., Atlanta Med. Col. ...................... 1
Alabama, Med. Assoc. of....... 88
*Trans*........ 444
Albumen, new property of...... 495
Albuminuria, treatment of...... 14
Alcohol food? Is. ............ 190
ALEXANDER, LAURENCE.....352, 456
ALEXANDER, L. B............... 591
American Medical Association.. 219
Ammonia, carbozotate of....... 433
Anæsthetics, mixed............ 152
Anæsthesia, sexual impressions during...................... 468
*Anatomy, Surgical;* BELLAMY... 53
Aneurism of femoral artery..... 513
Surgical treatment of....... 163
ANSTIE, FRANCIS (Obituary)..... 482
Anus, fissure of............... 432
Imperforate, with no rectum 461
Army Medical Corps........... 574
*Arteries, ligation of;* FARABEUF.. 311
Arteries, torsion of............. 337
Artery common iliac, immediate compression of........... 36
Femoral, aneurism of...... 513
Asthma, spasmodic, bel'ad'na in 466
Associations, Our State......... 250
Atlanta Academy Medicine, proceedings of...14, 76, 125, 145, 206
288, 458, 527, 673, 738
Atlanta Medical College........ 1
Auscultation, remarks on....... 81

BAKER, THOMAS H............... 10
BATTEY, ROBERT.......129, 734, 743
Belladonna in opium poisoning. 403
In spasmodic asthma....... 466
Bibliography...50, 117, 188, 245, 311
376, 444, 498, 569, 635, 700, 762
Bi-lateral operation for stone.... 425
Bismuth subnit., adulterated.... 631
BLACKBURN, LUKE P............ 405
Bladder, drainage in chronic inflammation of................ 297
Atony of ............. 675, 683
Blood-clots in vessels, deportment of....................... 385
Blood, drinkers of............. 465
Boots and shoes, maladies produced by..................... 438
Brain, gunshot wound of....... 747
The, its relations.......... 583
Infl'mat'n of membranes of.. 77
Physiology of. ............ 442
Brain-power of man............ 1[illegible]8
BURGESS, WM. R............... 1
Burns, new treatment of........ 374
Business, kings and slaves of... 539
Butter, artificial............... 171

Calculus, thirty-seven cases of urinary...................... 415
CALHOUN, A. W., 22, 65, 193, 330, 600
Calomel in functional hepatic diseases...................... 110
Saccharated ....61, 75, 277, 288
Camphor, mono-bromide of.... 497
Cancer, chloral in...........497, 561
Cures (formulæ)........... 299
Of foot.................... 524
Hereditary character of .... 374
Of stomach, bowels and omentum................ 200
Treatment of.............. 479
Of uterus.................. 45
Cane, The Goldheaded......... 255
Carbuncle, local treatment of... 365
Castor beans, cultivation of..... 559
Catalepsy with dysmenorrhœa.. 612
Cataract, cases of ...23, 26, 604, 605
Twenty-seven operations for 330
Catarrh remedy, Sage's ........ 365
Snuff...................... 568
Change, recreative............. 362
Child-bearing, limit of......... 624
*Children, Diseases of;* MEIGS and PEPPER.................... 53
Chloral, hydrate of..........73, 214
In trismus neontarum...... 489
Chloroform and ether.......... 485
Hypodermically........... 486
Influence of, on fœtus..485, 699
Narcosis .................. 464
In puerperal hæmorrhage .. 454
Resuscitation from.....363, 407
In strychnine poisoning.... 465
Chloroformization, pupil in.... 488
*Cholera;* MURRAY............... 249
Cholera, sporadic malignant ... 749
Chorea, queries on............. 704
Treatment of.............. 484
Cider, to keep sweet........... 568

Cinchona alkaloids, action on bacteria.................... 36
Clinic Eye and Ear, Atlanta Med. Col.......................22, 600
Medical, Atlanta Med. Col.. 81
*Clinical Lectures;* DAVIS......... 571
Coccydynia........................ 241
Cold. "catching"..........174, 753
Powder.................... 634
Colic, spermatic................ 555
Cologne, golden Farina........ 368
Colotomy, lumbar.............. 308
Coma, treatment of............ 458
Compression, digital, in femoral aneurism...................... 513
Immediate of common iliac 36
Confederate-ex Surgeons 126,182, 704
*Conservative Medicine, etc.;* FLINT 378
*Conspectus Med. Sci's;* LINCOLN.. 378
Constipation, excessive ........ 472
Consultations in the country ... 164
Coryza.......................... 101
COTTEN, J. F.................... 731
Cough, experimental study of .. 566
Coughs, to check............... 487
COUVERT, A. ................... 286
Crab-Orchard salts............. 405
Cranium, compound fracture of, 204
Cremation ..................124, 754
Cases of.................... 561
Elegy...................... 95
CROMWELL, BENJAMIN M........ 577
Croton chloral hydrate.....304, 400
*Croup and Tracheotomy;* COHEN.. 506
*Cyclopædia of Medicine;* VON ZIEMSSEN ....................... 635

Darwinism vs. Medicine ....... 620
In the kitchen.............. 684
Death, diagnosis of............ 760
Delirium, management of ...... 750
Tremens............99, 100, 106
*Dental Pathology;* SALTER....... 701
DEWITT, W. F................... 142
Diabetes ...................... 78
Diagnosis, surgical ........... 103
Diarrhœa...................... 43
Digitalis, action of..........16, 18
Diphtheria................731, 738
And scarlatina, correlation between ...........460, 738
Dislocations old, reduction of.. 288
Diuretics, action of........... 15
Doctors, London ............... 355
Douche nasal, use of.......... 19
Dysentery, ergot in........... 487
Iced-water enemata in ..... 256
Ipecacuanha in....291, 529, 659
Dysmenorrhœa ............641, 682
Gossypium in............... 484
With catalepsy............. 612
Dyspepsia................467, 748

Ear, removal of foreign bodies from....................631, 758
"Eclectic" obstetrics.......... 203
Eczema....................... 101
Capitis.................... 488
Editorial, 58, 124, 190, 250, 314, 383, 448, 508, 572, 636, 704, 764
Electricity, therapeutics of...... 746
*Electricity;* BEARD and ROCKWELL 700
*Electricity Static;* ARTHIUS...... 121
*Electrology and Neurology, Archives* ....................... 188
Electro-Medical Humbug ...... 697
*Electro-Therapeutics;* LINCOLN .. 378
*Elevations, etc., Dictionary of;* TONER ....................... 120
Elixirs standard.............. 236
Embolism..................... 309
Embryotomy.................. 572
Emulsions ................... 529
Epilepsy trephining for, case of, 193
Epistaxis, treatment of......... 683
Ergot, hypodermic injection of. 622
Erysipelas, treatment of, 678, 679 761
Esmarch's bloodless operation, 47, 526, 617
*Essentials of Medicine;* HARTSHORNE ...................... 502
Ether in labor................. 496
Ethics, medical........463, 479, 509
Euthanasia.................... 367
Eye and Ear Clinic Atlanta Medical College...............22, 600
Eyelids, granular...........99, 603

Fæces impacted................ 748
Fees, medical...............50, 764
Fever, cerebro-spinal.......... 751
Hay, Helmholtz's treatment. 431
Hæmorrhagic malarial or malignant bilious.....142, 214
340, 575, 591
Intermittent............... 100
Fistula, vesico-vaginal......... 176
Florida South, Medical Assoc'n. 383
Fœtus, deformities of.......... 71
Effect of chloroform on.485, 689
*Food and Dietetics;* PAVY....... 378
FOSTER, EUGENE............... 722
Fractures, multiple............ 677

Gallstones, solvent for.......... 557
Gelseminum, therapeutics of..48, 62
*Genito-Urinary Organs and Syphilis;* VAN BUREN............. 244
Georgia, laws of as to practice of medicine ...............10, 128
Medical Association of..... 84
*Trans*...... 380
Southwestern Med. Asso. of. 254
GILMORE, J. T..............257, 321
Gleet should a man with, marry? 624

Glossitis acute..............218, 353

Goitre cystic, treatment of...... 440

Gonorrhœa chronic, treatment of 480

Silicate of soda in.......... 366

Guiacum in acute tonsilitis..... 555

Hæmatocele................... 102
Hæmorrhage, post-partum...... 106
Chloroform in. 454
Hæmorrhoids, carbolic acid in.. 461
Harelip, new method for........ 613
HART, C. C.................... 203
HAUSER, WILLIAM.............. 404
Headache, aconite in........... 442
Guarana in................ 476
Hernia reduced by distension... 475
Strangulated, tractile method in.................. 305
*Homeopathy;* HEMPEL.......... 246
HOPKINS, T. S................. 199
Hydrometra, cases of........... 532
Hymen unruptured, deliv'y with. 457

Impetigo ..................... 493
Impotence from stricture....... 207
*Infancy and Childhood, Diseases of;* WEST.................. 377
*Infant Diet;* JACOBI ........... 446
Injection of castor oil vomited.. 492
Intestines, invagination of...... 486
Water in obstruction of ... 129, 208, 314
Intussusception, case of........ 598
Ipecacuanha, non-emetic use of. 659, 705
Remarks on. 674
Iridectomy, cases of......22, 24, 26

Jarborandi.................... 301
Jaundice, pathology of......... 624
JOHNSON, JOHN M..........277, 641
JOHNSON, WM. J............... 340

Kentucky Medical Society...... 93
Kitchen, Medicine of the....... 35

Labor, expedient in difficult.... 759
Laborer is worthy of his hire... 636
Lactation in advanced life...... 199
Lacto-Peptine................. 456
Laws of Georgia as to practice of medicine.............10, 128, 764
Of Missouri as to practice of medicine................ 367
LEWIS, JAMES O............353, 403
Lipoma....................... 522
Liver, functional derangem'ts of. 358
LOWE, J. H.................... 521
Lungs, local injection of cavities in..................108, 470

McCANN, JAMES................ 513
Maggots in external ear........ 74
Malformations congenital ...... 354
*Marine Hospital Service;* WOODWORTH...................... 121

*Maryland Med. Chir. Fac., Trans.* 381
*Materia Medica;* BIDDLE........ 312
*Medicine, Early American;* TONER 639
Medicine Georgia laws as to practice of.... ........10, 128, 764
State...................... 696
MEEK, J. A..............71, 74, 457
Menstruation, physiology of.... 350
Mercury, action of............ 742
In Syphilis................ 243
Milk, estimation of fat in ...... 761
In febrile diseases.......... 434
Suppression of, by mint.... 44
Milkleg, pathology and treatment of.................534, 606
Mimicry nervous .............. 161
Mind reading.................. 625
Mint, suppression of milk by... 44
Modern isms and ologies....... 481
MOONEY, M. D................. 354
Moonshine as medicine......... 436
MOORE, K. P.................. 200
Monster, double............154, 210
Montpellier oath............... 568

Neuralgia, camph'ated chloral in. 494
Neurotomy.................... 34
NOLAN, E. M.................. 73
Nomenclature, specimen of..... 698
*Obstetric Surgery;* CLAY........ 503
Occlusion of os uteri at term... 449
O'Flanagan, Dr................ 178
Oils, dangerous burning........ 435
Ophthalmia, gonorrhœal....... 602
Purulent.................. 601
Scrofulous........600, 601, 603
Sympathetic .............. 65
Opium poisoning, belladonna in 403
Oriental progress.............. 698
Os uteri, occlusion of at term... 449
OTT, I........................ 400
Ovaries, pathology of removed.. 160
Ovariotomy................... 147
Cases..................743, 747
Double, physiological results of.......................33, 364
Drainage in............... 368
Normal.................... 350
Normal, cases ......292, 321 685
Its relations to septicæmia, pyæmia, etc............. 257
Vaginal................... 145
*Ovariotomy, Retrospect of;* ATLEE 762
Ovary, physiology and pathology of the.............27, 351, 478
Ovulation and menstruation 208, 294
OWEN, GORONWY............... 449
Ozone, relations of to disease... 474

Paracentesis thoracis........... 45
Patella, fracture of............. 155
Pepsin in diphtheria........... 443
Perineum ruptured, cases...... 734
Peritomy..................... 676

*Pharmacy;* PARRISH............ 376
Phlegmasia dolens......79, 534, 606
*Physical Meas'm'ts,* KOHLRAUSCH 50
Physician and God of Love .... 616
Physicians consulting, legal responsibility of............... 171
Phytolacca in mastitis.......... 352
To kill cockroaches........ 373
Piles, removal of.............. 156
Pot? Is there Death in the...... 58
Potassa chlorate, explosion of .. 489
Potassium iodide, admin'tion of 448
Potio dura, paralysis of........ 290
Powder stain, bread in......... 614
Pregnancy, extra-uterine 79, 527, 606
Prescriptions.................. 480
PROCTER, JR., WM., (obituary).. 63
Prostate, ergot in enlarged..... 460
Pruritus vulvæ, treatment of.... 557
*Puerperal Diseases;* BARKER .... 51

Quickening as a diagnostic sign, 216
Quinia sulphate, pills of........ 480
Therapeutic action of...... 760
Quinine a cardiac sedative...... 698
Pill mass .................. 300
In whooping-cough........ 44

Rain, some things about........ 627
RAMSEY, FRANK A............... 385
RAUSCHENBURG, CH............... 350
Rectum, delivery through the.. 449
Registration of vital statistics... 637
Regurgitation of food, habitual.. 113
Rheumatism, statistics of....... 174
Rigor puerperal, explanation of. 761
Ring, gold or brass, to remove.. 404
RUDICIL, R. Y.................. 204
Rusticus in town............... 285

Salts, Crab-Orchard............ 405
SAXON, W. H.................... 288
Scopemania..... .. ............ 689
SIMS, HARRY L.................. 337
SIMS, J. MARION................ 4[illegible]7
Skin, absorption by............ 752
Skull, compound fracture of.... 204
SMITH, B. C.................... 598
Smoking a sin? Is...... ..... 556
*So. Carolina Med. Asso., Trans..* 380
Specialties, college for......... 302
Speculum, Erich's vaginal...... 428
Spermatorrhœa................ 100
Spleen, dislocation of..211, 613, 632
Excision of................ 762
Hypertrophy, Brom. Pot. in 757
*Sphygmograph, The;* HOLDEN.... 51
Stair climbing, female dis. from 469
Stammering .................... 565
STANFORD, F. A ............... 425
Staphyloma ................... 604
STARR, E. F.................... 583
State Medicine ................ 696
Sterility a result of lithotomy... 443
Stomach, washing out the...... 477
Stone, bi-lateral operation for... 425
Stricture, impermeable, 216, 615, 673
Manipulation in........... 483
Strychnia in whiskey.......... 303
*Surgical Emergencies;* SWAIN.... 505
Syphilis, notes on.............. 46
Treatment of.............. 680
Treatment of true.......... 722
Tape-worm, ether for.......... 492
New treatment for.......... 494
Teeth deciduous, extraction of.. 366
Temperature in surgical cases.. 48
Lowest..................... 495
Tendo-Achilis, division of...... 371
Silver sutures in........... 372
Tennessee Medical Society...... 90
Tents sponge, death following 369
Testicle inflamed, treatment of.. 530
*Therapeutics;* STILLE........... 569
*Therapeutics;* H. C. WOOD...... 117
Thought modern............... 96
Toe-nails inverted, treatment of. 432
Tongue, acute inflam't'n of..218, 353
Torsion of arteries............. 337
*Toxicology;* REESE............. 120
Tracheotomy................... 167
Transfusion..... ........234, 621
Of lamb's blood............ 366
Trephining for epilepsy, case .. 193
Tumors dispersed by puncture.. 475

Ulcers old, new application for. 368
Uræmia, induction of labor, 116, 170
Urethrotomy.................. 166
*Urinary Organs;* THOMPSON..... 571
*Urine, Examination of;* TYSON... 571
Urine, improved test for sugar in 623
Uterine-intra medication....... 235
Stem ............ 290
Uterus, ante-version of......... 521
Cauterization of........427, 558
Removal of tumor from.... 286
Rudimentary.............. 207
Ulceration of.............. 174

Vagina, chronic inflammation.. 310
Injections of hot water in.. 473
VERDERY, P. S................. 454
Vertigo as a brain symptom.... 756
Vice, hereditary.... .......... 759
*Virginia Medical Society Trans..* 122
Vitreous, opacity of............ 25
Vomiting, Bro. Pot. in obstinate 479

WESTMORELAND, J. G. ......... 81
WESTMORELAND, W. F......415, 522
Whooping-cough.......44, 634, 760
Novel remedy for.......... 320
"Wolf" in child's scalp........ 21
*Women, Diseases of;* BARNES.... 54
*Women, Diseases of;* THOMAS.... 498
WOODHULL, ALFRED A. ......659, 705

*Yellow Fever, Urine in;* JONES... 57

Zinc oxide of, in diarrhœa...... 43

